# Do biologic therapies reduce aortic inflammation in rheumatoid arthritis patients?

**DOI:** 10.1186/s13075-021-02585-w

**Published:** 2021-08-03

**Authors:** D. A. M. Thuy Trang, Koichi Okamura, Takahito Suto, Hideo Sakane, Yukio Yonemoto, Takahito Nakajima, Yoshito Tsushima, Hirotaka Chikuda

**Affiliations:** 1 Department of Orthopaedic Surgery, Gunma University Graduate School of Medicine, Showamachi 3-39-15, Maebashi, Gunma 371-8511 Japan; 2 Department of Diagnostic Radiology and Nuclear Medicine, Gunma University Graduate School of Medicine, Showa-machi 3-39-15, Maebashi, Gunma 371-8511 Japan; 3 Radiology Center, Bach Mai Hospital, Hanoi, Vietnam; 4 Department of Diagnostic Radiology and Interventional Radiology, Tsukuba University, Tsukuba, Ibaraki, Japan; 5 Research Program for Diagnostic and Molecular Imaging, Division of Integrated Oncology Research, Gunma University Initiative for Advanced Research (GIAR), Maebashi, Gunma Japan

**Keywords:** Aortic inflammation, FDG PET/CT, Biologic therapy, Rheumatoid arthritis

## Abstract

**Objectives:**

Rheumatoid arthritis (RA) patients have an increased risk of cardiovascular disease (CVD). In the present study, we evaluated the inflammatory activity of the ascending aorta in RA patients who received biological treatment.

**Methods:**

We assessed the aortic wall inflammation of RA patients using ^18^F-fluorodeoxyglucose (FDG) positron emission tomography/computed tomography before and after 6 months of biologic therapies. We also compared the inflammatory activity at the aortic wall in RA patients with remission or low disease activity (RLDA) and those with moderate or high disease activity (MHDA). The aortic uptake was measured by the standardized uptake value (SUV) and the target-to-background ratio (TBR).

**Results:**

A total of 64 patients were included in the analysis (mean age, 58.4 ± 13.8 years old; female, 77%). The Disease Activity Score for 28 joints (DAS28) erythrocyte sedimentation rate (ESR) had significantly decreased after 6 months: from 5.0 ± 1.2 to 3.3 ± 1.2 (*p* < 0.001). The FDG uptake in the ascending aorta changed from baseline to 6 months, showing a maximum SUV (SUV_max_) of 1.83 ± 0.34 to 1.90 ± 0.34 (*p* = 0.059) and TBR of 1.71 ± 0.23 to 1.75 ± 0.24 (*p* = 0.222). The SUV_max_ and TBR after 6 months were significantly higher in the RLDA group than in the MHDA group (2.05 ± 0.32 vs. 1.79 ± 0.33 (*p* = 0.002) and 1.89 ± 0.33 vs. 1.65 ± 0.20 (*p* = 0.001), respectively). The percentage of monocytes also significantly increased from baseline to 6 months: from 5.9 ± 1.6 to 6.9 ± 2.6 (*p* = 0.032).

**Conclusion:**

The inflammation activity at the ascending aorta in RA patients did not change significantly after 6 months of biological treatment. RA patients with a low disease activity or in clinical remission after 6 months of biological treatment still had an increased inflammatory activity at the aortic wall.

## Background

Rheumatoid arthritis (RA) increases the risk of cardiovascular disease (CVD) [1–3]. An accelerated progression of atherosclerosis leads to an increased mortality in RA patients [4–10]. Because both an inflamed synovial membrane and atherosclerotic plaque share important common pathological processes, chronic systemic inflammation might accelerate the development of atherosclerosis in RA patients [10]. Previous studies have demonstrated that atherosclerosis is more prevalent in the RA population than in healthy individuals [4–9].

^18^F-fluorodeoxyglucose (FDG) positron emission tomography (PET)/computed tomography (CT) can be used to evaluate the joints in RA patients [11–16]. In addition, FDG PET/CT has also been widely used to evaluate atherosclerosis, since the ^18^F-FDG uptake reflects the glucose metabolism of macrophages in atherosclerotic plaque [17–21]. However, while FDG PET/CT is a highly reproducible method of evaluating arterial inflammation, there have been few reports regarding its utility in assessing aortic inflammation in RA patients.

In the present study, we assessed the ascending aortic wall inflammation in RA patients who received biologic therapies using ^18^F-FDG PET/CT. We also compared the inflammatory activity at the aortic wall in RA patients in remission or with a low disease activity (RLDA) and those with moderate or high disease activity (MHDA) after biologic treatment.

## Materials and methods

### Study population

Sixty-four RA patients who underwent whole-body ^18^F-FDG PET/CT at baseline and 6 months after the initiation of biologic therapies at Gunma University Hospital were enrolled in this study. All patients were diagnosed with RA according to the American College of Rheumatology criteria revised in 1987 and had a history of inadequate clinical response to previous treatments with nonbiological disease-modifying antirheumatic drugs (DMARDs), such as methotrexate (MTX). Therefore, these patients had been recommended for treatment with biologics.

The study protocol was approved by the Institutional Review Board of Gunma University Hospital. Written, informed consent was obtained from each patient before they participated in the study.

### Imaging acquisition

Whole-body ^18^F-FDG PET/CT was performed using a PET/CT scanner (Biograph 16; Siemens Medical Solutions Inc., Malvern, PA, USA) before and 6 months after the initiation of biologic therapies. In brief, ^18^F-FDG (5 MBq/kg) was injected intravenously after at least 6 h of fasting. Patients were imaged approximately 60 min after FDG injection in the supine position. A non-contrast-enhanced CT scan (140 kV, 120–240 mAs) for attenuation correction and anatomic co-registration was obtained before PET imaging. PET imaging was performed in the 3-dimensional mode with 3 min per bed position and slice thickness of 3.27 mm. The PET images were reconstructed using an ordered-subsets expectation-maximization algorithm into 128 × 128 matrices as previously described [11, 13].

### Imaging analyses

Image analyses were performed using the *syngo.*via software program (Siemens Healthcare, Erlangen, Germany). An independent radiologist blinded to the clinical data analyzed all of the PET/CT images. According to the recommendation of the Cardiovascular Committee of the European Association of Nuclear Medicine (EANM) [22], we used the maximum standardized uptake value (SUV_max_) and the target-to-background ratio (TBR) as parameters for the FDG uptake in the ascending aorta. The SUV_max_ and TBR correlate with histological markers of inflammation and have been used in several studies to measure the FDG uptake in the arterial wall [17, 18, 23, 24].

Ascending aorta inflammation was quantified within each region of interest (ROI), containing the arterial wall and the lumen, along the length of the ascending aorta. The ROIs were manually drawn every 5 mm on axial images, starting 1 cm above the origin of the left main coronary artery and ending at the merging point with the aortic arch. The SUV is determined as the radioactivity concentration (kBq/ml) within an ROI divided by the decay-corrected amount of injected dose per patient’s weight (kBq/g). The average of the SUV_max_ (mean SUV_max_) for all ROIs of the entire ascending aorta was then calculated. Subsequently, the TBR was obtained as the mean SUV_max_ divided by the background SUV_max_ in the superior vena cava to correct for the blood activity.

### Clinical and laboratory evaluations

RA disease activity was assessed using the Disease Activity Score for 28 joints (DAS28) based on the ESR at baseline and 6 months after the initiation of biologic therapies. We also measured the white blood cell (WBC) count, serum levels of erythrocyte sedimentation rate (ESR), C-reactive protein (CRP), matrix metalloproteinase-3 (MMP-3), anti-cyclic citrullinated peptide antibodies (ACPA), and rheumatoid factor antibodies (RF).

The patients were divided into 2 groups according to the DAS28-ESR at 6 months: RLDA in those with a DAS28-ESR < 3.2 and MHDA in those with a DAS28-ESR ≥ 3.2. The clinical parameters and FDG uptake for the ascending aorta at baseline were compared between these two groups.

### Statistical analyses

Data analyses were performed using the IBM SPSS Statistics 25 software program (IBM Corp., Armonk, NY, USA). Data were expressed as the mean ± standard deviation for parametric variables, as the median and interquartile range for nonparametric variables, and as numbers and percentages for categorical variables. For the comparison of continuous data between two clinical response groups, an unpaired Student’s *t*-test was used. For the comparison of continuous data before and after treatment, paired Student’s *t*-test or Wilcoxon’s signed-rank test was used. For the comparison of categorical data, the chi-square test or Fisher’s exact test was used. A value of *p* < 0.05 was considered statistically significant.

## Results

**Fig. 1:**
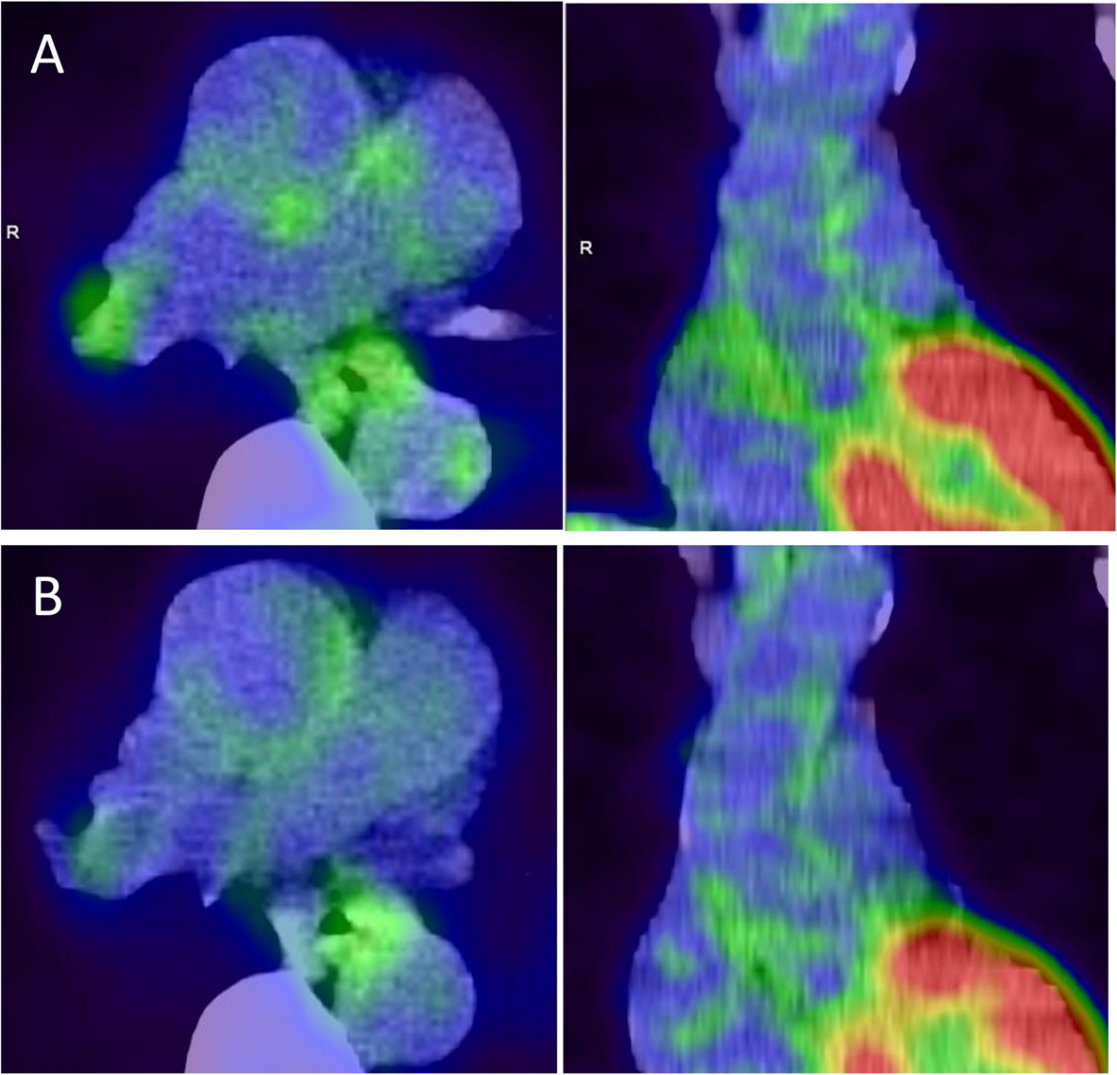
Typical ^18^F-FDG PET/CT images of the ascending aorta wall uptake at baseline and after 6 months of biologic therapy. Axial and coronal images of ascending aorta from a 65-year-old female patient who underwent treatment with infliximab. **A** At baseline, the mean SUV_max_ was 1.64, and the TBR was 1.63. **B** At 6 months, the mean SUV_max_ was 1.56, and the TBR was 1.44. SUV_max_, maximum standardized uptake value; TBR, target-to-background ratio

A total of 64 patients were included in the analysis (mean age, 58.4 ± 13.8 years old; females, 77%) (Table 1). The mean disease duration was 13.1 ± 11.8 years. A total of 70% of the patients received MTX, and 48% received prednisolone (PSL). Figure 1 shows the ^18^F-FDG PET/CT findings for a case at baseline and 6 months after the initiation of biologic therapy. The mean FDG uptake values for the ascending aorta and the clinical parameters at baseline and 6 months after the initiation of biologic treatments are shown in Table 2. Although all clinical parameters except for the ACPA were significantly decreased at 6 months compared with the baseline, the mean SUV_max_ and TBR were not significantly changed after 6 months (Table 2).

**Table 1:** The demographic characteristics and medication use of patients at baseline

Clinical characteristics	Values
Age (years)	58.4 ± 13.8
Female/male	49/15
Disease duration (years)	13.1 ± 11.8
BMI	21.8 ± 4.0
Smoking, *n* (%)	8 (12.7)
Hypertension, *n* (%)	23 (36.5)
Diabetes, *n* (%)	6 (9.7)
Total cholesterol (mg/dL)	197.4 ± 39.3
LDL (mg/dL)	114.9 ± 37.5
HDL (mg/dL)	59.6 ± 15.0
Triglycerides (mg/dL)	108.2 ± 46.0
PSL use, *n* (%)	31 (48.4)
PSL dose (mg/day)	2.0 ± 2.3
MTX use, *n* (%)	45 (70.3)
MTX dose (mg/week)	5.7 ± 3.5
Biologics (IFX/ETN/ADA/GLM/TCZ/ABT)	18/14/16/2/13/1

The data are expressed as the mean ± standard deviation

*BMI* body mass index; *LDL* low-density lipoprotein cholesterol; *HDL* high-density lipoprotein cholesterol; *PSL* prednisolone; *MTX* methotrexate; *IFX* infliximab; *ETN* etanercept; *ADA* adalimumab; *GLM* golimumab; *TCZ* tocilizumab; *ABT* abatacept

**Table 2:** Changes in FDG parameters and clinical parameters after treatment

Parameters	Baseline	After 6 months	***p*** value
FDG parameters			
Mean SUV_max_	1.83 ± 0.34	1.90 ± 0.34	0.059
TBR	1.71 ± 0.23	1.75 ± 0.29	0.222
Clinical parameters			
ESR (mm/h)	62.3 ± 32.2	36.5 ± 28.6	< 0.001
CRP (mg/dL)^a^	1.3 (0.4–3.0)	0.1 (0.0–0.6)	< 0.001
MMP-3 (ng/mL)^a^	153.6 (70.5–412.6)	65.2 (39.3–133.4)	< 0.001
WBC (/μL)	6537.5 ± 2239.2	5609.4 ± 1992.5	< 0.001
% neutrophils	68.7 ± 9.2	57.9 ± 12.7	< 0.001
% eosinophils	2.3 ± 2.3	3.0 ± 2.6	0.004
% basophils	0.4 ± 0.2	0.5 ± 0.3	< 0.001
% monocytes	5.3 ± 1.8	6.3 ± 2.2	0.003
% lymphocytes	22.2 ± 7.5	31.3 ± 11.6	< 0.001
ACPA (U/mL)^a^	87.4 (12.1–100.0)	55.8 (8.0–100.0)	0.276
ACPA positive, *n* (%)	34 (53.1)	29 (45.3)	< 0.001
RF (U/mL)^a^	49 (13–137)	27 (10–79)	< 0.001
RF positive, *n* (%)	34 (53.1)	25 (39.1)	< 0.001
DAS28-ESR	5.0 ± 1.2	3.3 ± 1.2	< 0.001

The data are expressed as the mean ± standard deviation, with the exception of skewed variables (^a^), which are represented as medians (interquartile range)

*SUV*_*max*_ maximum standardized uptake value; *TBR* tissue-to-background ratio; *ESR* erythrocyte sedimentation rate; *CRP* C-reactive protein; *WBC* white blood cell count; *MMP-3* matrix metalloproteinase-3; *ACPA* anti-cyclic citrullinated peptide antibody; *RF* rheumatoid factor; *DAS28* Disease Activity Score in 28 joints

**Fig. 2:**
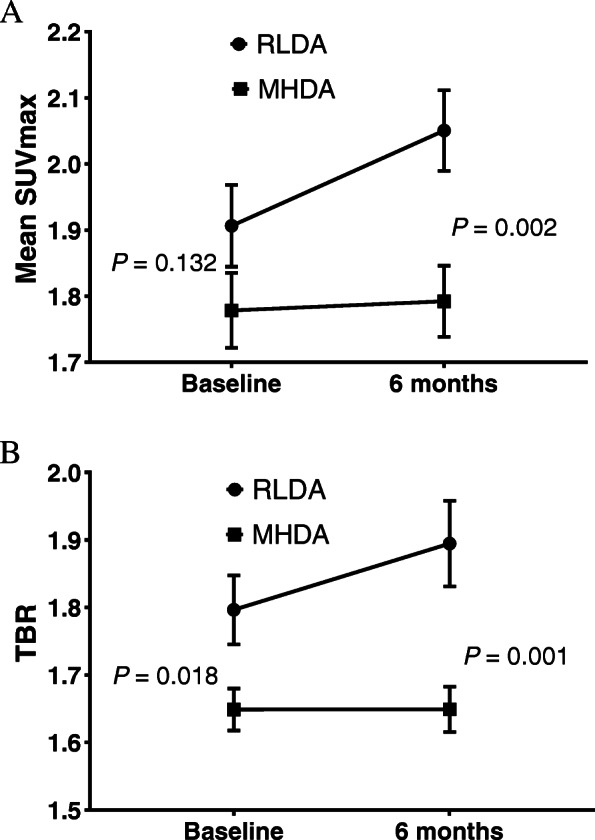
FDG uptake parameters of the ascending aorta for the two clinical response groups. The mean SUV_max_ and TBR values of the ascending aorta at 6 months after biologic treatment were significantly higher in the group with a DAS28-ESR < 3.2 (indicating remission or low disease activity) than in the group with a DAS28-ESR ≥ 3.2 (indicating moderate or high disease activity). SUV_max_, maximum standardized uptake value; TBR, target-to-background ratio; DAS28, Disease Activity Score in 28 joints; RLDA, remission or low disease activity; MHDA, moderate or high disease activity

We compared the FDG uptake at the ascending aorta between the two groups (RLDA group vs. MHDA group) at baseline and after 6 months of treatment. Figure 2 shows the increases in the mean SUV_max_ and TBR values for the ascending aorta at 6 months after the initiation of treatment, especially in the RLDA group. The mean SUV_max_ value at 6 months was significantly higher in the RLDA group than in the MHDA group (2.05 ± 0.32 vs. 1.79 ± 0.33; *p* = 0.002). The TBR value at baseline was significantly higher in the RLDA group than in the MHDA group (1.79 ± 0.26 vs. 1.65 ± 0.19; *p* = 0.018). After 6 months, the TBR value was also significantly higher in the RLDA group than in the MHDA group (1.89 ± 0.33 vs. 1.65 ± 0.20; *p* = 0.001).

At baseline, the WBC count and percentage of monocytes were significantly higher in the RLDA group than in the MHDA group (*p* = 0.005 and *p* = 0.035) (Table 3). Regarding the WBC subtypes at 6 months, there were significantly lower percentages of neutrophils (*p* < 0.001) and significantly higher percentages of lymphocytes (*p* < 0.001) in the RLDA group than in the MHDA group (Table 4). The percentage of monocytes significantly increased from baseline to 6 months (5.9 ± 1.6 to 6.9 ± 2.6; *p* = 0.032).

**Table 3:** The comparison of clinical parameters at baseline

	RLDA (***n*** = 27)	MHDA (***n*** = 37)	***p*** value
Age (years)	56.3 ± 15.7	59.9 ± 12.1	0.334
Female/male	18/9	31/6	0.110
Disease duration (years)	10.3 ± 8.3	15.1 ± 13.6	0.082
PSL dose (mg/day)	2.2 ± 2.5	1.8 ± 2.1	0.483
MTX dose (mg/week)	5.2 ± 3.4	6.1 ± 3.6	0.321
ESR (mm/h)	53.1 ± 33.9	68.9 ± 29.5	0.056
CRP (mg/dL)^a^	1.7 (0.4–3.1)	1.3 (0.6–2.9)	0.807
WBC (/μL)	7429.6 ± 2078.1	5886.5 ± 2150.2	**0.005**
% neutrophils	68.3 ± 9.2	69.0 ± 9.3	0.775
% eosinophils	2.0 ± 1.1	2.6 ± 2.8	0.418
% basophils	0.4 ± 0.2	0.4 ± 0.2	0.699
% monocytes	5.9 ± 1.6	4.9 ± 1.9	**0.035**
% lymphocytes	22.4 ± 7.9	22.0 ± 7.3	0.857
MMP-3 (ng/mL)^a^	158.0 (95.0–399.3)	134.5 (69.1–500.0)	0.579
ACPA (U/mL)^a^	61.4 (10.7–100.0)	99.1 (15.1–100.0)	0.393
ACPA ≥ 60 U/mL, *n* (%)	13 (50.0)	21 (58.3)	0.515
RF (U/mL)^a^	33 (10–100)	61.5 (23.8–211.8)	0.082
RF ≥ 40 U/mL, *n* (%)	12 (48.0)	22 (64.7)	0.199
DAS28-ESR	4.5 ± 1.4	5.3 ± 1.0	**0.008**

The data are expressed as the mean ± standard deviation, with the exception of skewed variables (^a^), which are represented as medians (interquartile range)

*RLDA* remission and low disease activity; *MHDA* moderate and high disease activity; *PSL* prednisolone; *MTX* methotrexate; *ESR* erythrocyte sedimentation rate; *CRP* C-reactive protein; *WBC* white blood cell count; *MMP-3* matrix metalloproteinase-3; *ACPA* anti-cyclic citrullinated peptide antibody; *RF* rheumatoid factor; *DAS28* Disease Activity Score in 28 joints

**Table 4:** Counts for WBC subtypes at 6 months in the two groups

	RLDA (***n*** = 27)	MHDA (***n*** = 37)	***p*** value
WBC (/μL)	5807.4 ± 1994.2	5465.0 ± 2006.1	0.501
% neutrophils	51.3 ± 12.0	62.8 ± 11.0	**< 0.001**
% eosinophils	2.8 ± 2.0	3.0 ± 2.9	0.881
% basophils	0.6 ± 0.4	0.4 ± 0.2	0.114
% monocytes	6.9 ± 2.6	5.8 ± 1.7	0.065
% lymphocytes	37.7 ± 11.3	26.7 ± 9.5	**< 0.001**

The data are expressed as the mean ± standard deviation

*RLDA* remission and low disease activity; *MHDA* moderate and high disease activity; *WBC* white blood cell

We compared the FDG uptake and clinical parameters between the groups with and without anti-tumor necrosis factor (TNF) drug treatment, but there were no significant differences between these two groups (data not shown).

## Discussion

In the present study, we found that the inflammation activity of the ascending aorta in RA patients did not significantly change after 6 months of biologic treatment. RA patients with RLDA after 6 months had a significantly higher FDG uptake at the aortic wall than those with MDLA.

Biological agents showed beneficial effects on reducing RA symptoms and disease activity by suppressing inflammation. Since atherosclerosis is a chronic inflammation process, these drugs may be also effective in reducing CVD risk in RA. Previous reports have shown that 8-week anti-TNF-α therapy reduced aortic inflammation in RA patients [25]. However, the FDG uptake of the arterial wall after treatment was still significantly higher in RA patients than in the control group.

In our study, although the disease activity and serum markers of RA patients significantly decreased after 6 months of biologic treatment, the inflammatory activity of the ascending aorta still existed. This result indicated that the cardio-protective effects of biologic therapy might not apply to all RA patients at six months.

A cross-sectional study in the USA demonstrated that RA patients who needed anti-TNF therapy to continue their clinical remission had a greater FDG uptake at the ascending aorta than those receiving non-biological DMARDs [26]. In addition to the high disease activity before the biological treatment, our patients had a long disease duration after the onset of RA. These patients’ condition might have led to the remnant aortic inflammation after 6 months of biological treatment, even if their joints had a good clinical response to biological drugs. Our results indicated that if we were intending to use biological drugs to achieve a cardio-protective effect, we should ensure RA patients maintain a low disease activity for a long time.

The FDG uptake in the aortic wall was significantly higher in RA patients with RLDA than in those with MHDA at 6 months. This was a controversial result. However, as mentioned above, our follow-up period was only 6 months and thought to be insufficient to reduce systemic inflammation by RA. It is thus possible that a longer treatment period led to the reduced aortic inflammation in the RLDA group.

In addition, a 3-year, prospective, observational study in an Italian cohort clearly showed that the maintenance of remission was associated with a reduced risk of atherosclerosis [27]. Our results showed that while the use of biologic therapies had reduced the clinical symptoms at 6 months, the aortic wall inflammation in these patients persisted despite 6 months of biologic treatment. Therefore, tight control of the RA activity in these patients should be maintained, and their CVD risk should be carefully estimated.

In this study, we also noted an elevation of monocytes at baseline and at 6 months in the RLDA group. An increased activation of circulating monocytes has been reported in patients receiving anti-TNF therapy [26] and the elevations of monocyte subpopulation can be seen in RA patients with coronary artery atherosclerosis and are associated with an increased CVD risk [28, 29]. Furthermore, a previous in vivo imaging study reported that the peripheral blood mononuclear cell accumulation was correlated with the arterial wall inflammation assessed on hybrid single-photon emission computed tomography/CT [30]. These previous findings suggest that the increased percentage of monocytes might be involved in arterial wall inflammation in RA patients, even after biologic therapy.

Several limitations associated with the present study warrant mention. First, traditional cardiovascular risk factors could not be fully investigated because of the lack of such information. Second, the selection of biologic agents was left to each physician. Since different drugs have different mechanisms through which they act on RA pathogenesis, treatment bias may exist. Third, since FDG-PET/CT is a nuclear imaging modality, repeated examinations might lead to increased radiation exposure. Finally, this was a short-term study. To determine whether or not biological therapies are useful for reducing aortic inflammation and CVD risk in RA patients, further long-term follow-up research should be conducted.

## Conclusions

We demonstrated that the inflammation activity of the ascending aorta in RA patients was not significantly altered after 6 months of biologic treatment. RA patients with a low disease activity or in clinical remission after 6 months of such treatment still had an increased inflammatory activity at the aortic wall.

## Data Availability

All data generated or analyzed during this study are included in this published article and its supplementary information file.

## Abbreviations

ACPA: Anti-cyclic citrullinated peptide antibody
CRP: C-reactive protein
DAS: Disease activity score
DMARDs: Disease-modifying antirheumatic drugs
CVD: Cardiovascular disease
EANM: European association of nuclear medicine
ESR: Erythrocyte sedimentation rate
FDG-PET/CT: Fluorodeoxyglucose-positron emission tomography/computed tomography
RLDA: RA patients in remission or with a low disease activity
MHDA: RA patients with moderate or high disease activity
MMP-3: Matrix metalloproteinase 3
MTX: Methotrexate
PSL: Prednisolone
RA: Rheumatoid arthritis
RF: Rheumatoid factor
ROI: Region of interest
SUV: Standardized uptake value
SUVmax: Maximum standardized uptake value
TBR: Target-to-background ratio
TNF: Tumor necrosis factor
WBC: White blood cell
